# Coupled Thermal and Nonlocal Effects in Advanced Composite Nanobeams: An Efficient Higher-Order Modeling Approach

**DOI:** 10.3390/nano16140852

**Published:** 2026-07-10

**Authors:** Rabab A. Alghanmi, Mohammed Sid Ahmed Houari

**Affiliations:** 1 Department of Mathematics, College of Sciences and Arts, King Abdulaziz University, Rabigh 21911, Saudi Arabia; raalghanmi@kau.edu.sa; 2 Laboratoire d’Etude des Structures et de Mécanique des Matériaux, Département de Génie Civil, Faculté des Sciences et de la Technologie, Université Mustapha Stambouli, B.P. 305, Mascara 29000, Algeria

**Keywords:** FG nanobeams, nonlocal elasticity, thermo-mechanical behavior, dynamic analysis, size-dependent effects

## Abstract

The present research proposes a mathematical method to examine the combined impacts of thermal loading and nonlocal elasticity on the dynamic behavior of functionally graded (FG) nanobeams. A refined higher-order shear deformation theory is introduced, featuring an enhanced displacement field with integral unknowns and a hyperbolic thickness-dependent function to accurately capture transverse shear strains without shear correction constants. Temperature affects the material characteristics, which follow a power-law pattern as they rise through thickness. Using Hamilton’s principle, the study establishes and solves the governing equations analytically. The study discusses how thermal environment, material gradation, and nonlocality alter the dynamic response of FG nanobeams, providing important insights into their nanoscale thermomechanical behavior.

## 1. Introduction

Functionally graded materials (FGMs) are innovative materials having characteristics that vary continuously across their structure, allowing for increased performance in demanding operating situations. These materials find extensive application in high-temperature engineering systems, including aerospace components, nuclear fusion reactor structures, and thermal protection coatings [1,2,3]. This category of materials addresses common issues found in laminated composites, such as matrix-fiber debonding, delamination, and matrix cracking [4]. Their graded nature ensures smooth stress distribution and improved resistance to thermal and mechanical loads. In recent decades, FGMs have attracted growing interest in the aerospace, marine, and automotive industries. Their ability to meet specific functional demands makes them ideal for next-generation engineering applications.

The primary advantage of FGMs is their continuous material variation, which avoids interfacial stresses and promotes smoother mechanical load transfer [5]. Nanoscale FGM applications benefit from their excellent thermomechanical properties, particularly in structural and functional nanomaterials [6,7,8].

Predicting the dynamic and mechanical properties of FG nanostructures constitutes a fundamental engineering challenge. Researchers employ numerical techniques, experimental procedures, analytical formulations, and molecular dynamics simulations to investigate these systems [9]. However, conducting experiments at the nanoscale remains highly complex, while molecular dynamics simulations are time-consuming and demand substantial computational resources. The need to understand FG nanostructure dynamics has driven international research into improved modeling techniques for micro/nano-system applications.

The development of materials capable of coupling two or more physical effects, such as electrical, magnetic, elastic, or thermal interactions, is essential for advancing modern engineering and manufacturing, particularly at the micro- and nanoscale. These multifunctional materials exhibit synergistic behaviors that significantly outperform those of conventional single-phase materials. They are especially critical in the design of micro- and nanostructured devices, including smart sensors, energy harvesters, and adaptive systems. By tailoring these coupled interactions, engineers can enhance the efficiency, durability, and responsiveness of next-generation technologies. Ultimately, such materials unlock new possibilities for multifunctional applications in fields like aerospace, biomedical engineering, and nano-electromechanical systems.

FGMs are extensively used in nanodevices like nanosensors and NEMS due to their exceptional environmental durability and mechanical performance at small scales. Their ceramic-metal graded composition provides outstanding wear resistance, load capacity, and thermal stability [10].

This project aims to design and develop an accurate analytical model that captures the simultaneous impact of thermal loading, nonlocal elasticity, and stretching on the dynamic reaction of the proposed nanostructure, providing a solid base for its application in advanced engineering systems and industrial structures.

Recent research demonstrates an increasing focus on both experimental and computational studies of FG structures’ mechanical and hygrothermal response. Given the difficulties in nanoscale experimental control, theoretical modeling has emerged as a vital analytical tool. At the nanoscale, size effects have an enormous impact on structural mechanical behavior. Therefore, accounting for these effects is essential for accurately analyzing and designing such small-scale systems. Since classical continuum mechanics lacks the ability to capture size-dependent phenomena, it is generally inadequate for modeling the behavior of nanostructures.

To overcome the limitations associated with classical continuum mechanics, a number of refined continuum models have been developed. Among these are the nonlocal elasticity theory [11,12], which introduces material length-scale impacts, the strain gradient theory [13,14,15], and the nonlocal strain gradient theory [16]. These advanced models enable a more accurate description of size-dependent behaviors in nanoscale structures.

Recent advances in size-dependent continuum mechanics have considerably improved the modeling and analysis of functionally graded (FG) nanobeams subjected to coupled thermo-mechanical environments. Within the framework of nonlocal strain gradient elasticity, Merzouki and Houari [17] developed an efficient finite element formulation for the thermal free vibration analysis of temperature-dependent FG nanobeams under uniform, linear, and nonlinear thermal loadings, demonstrating the combined influence of nonlocality, strain gradient effects, material gradation, and thermal environments on the dynamic response. Similarly, Penna et al. [18] proposed a local/nonlocal stress-gradient elasticity formulation to investigate the hygro-thermal vibration behavior of porous FG Bernoulli–Euler nanobeams, highlighting the important roles of porosity, material gradation, nonlocal interactions, and gradient length scales in accurately predicting nanoscale structural dynamics. More recently, Dang Van Hieu et al. [19] employed a refined shear deformation beam theory combined with Eringen’s nonlocal elasticity to analyze the hygro-thermal vibration of functionally graded carbon nanotube-reinforced composite (FG-CNTRC) nanobeams, emphasizing the effects of carbon nanotube distribution, moisture, temperature, beam theories, and nonlocal parameters on free vibration characteristics. In addition, Esen et al. [20] investigated the coupled free-vibration and buckling responses of temperature-dependent FG nanobeams subjected to magnetic and thermal fields using a nonlocal strain-gradient Timoshenko beam model, demonstrating the importance of simultaneously accounting for nonlocality, microstructural effects, thermal loading, and electromagnetic interactions. These studies clearly confirm that size-dependent continuum theories have become indispensable tools for accurately describing the thermo-mechanical behavior of nanostructures. Nevertheless, most of the available formulations are established on Bernoulli–Euler beam theory, Timoshenko beam theory, or conventional refined beam models, whose capability to accurately reproduce the transverse shear strain distribution remains dependent on the adopted kinematic assumptions and, in several cases, on the use of shear correction factors. Consequently, there is still a need for more efficient higher-order beam formulations capable of accurately representing transverse shear deformation while simultaneously accounting for temperature-dependent material properties and size-dependent nonlocal effects.

Recent studies have extensively explored the mechanical behavior of advanced nanobeams under various coupled physical fields using nonlocal elasticity theories. Najafi and Ahmadi [21] investigated the bending behavior of magneto-electro-elastic nanobeams, with particular attention to the effects of external electric and magnetic fields. Ren and Qing [22] examined the linear buckling and free vibration characteristics of FG piezoelectric beams within the framework of nonlocal integral elasticity theory. To handle complex loading scenarios in curved nanostructures, Pham et al. [23] established a two-node beam element that combines Lagrange and Hermite interpolations, applied to functionally graded porous nanobeams supported by an elastic base and exposed to hygrothermal and magnetic influences. Tran et al. [24] investigated the free vibration response of functionally graded Timoshenko nanobeams placed on a Winkler base, assuming nonlocal elasticity. In a similar study, Gartia and Chakraverty [25] analyzed the vibrational reaction of bidirectional FG nanobeams on Winkler\Pasternak foundations, emphasizing the significant impact of material gradation on dynamic performance. Ghazwani et al. [26] evaluated a high-frequency dynamic examination of FG sandwich nanobeams by means of a nonlocal quasi-3D approach in combination with Navier’s solution method. Uzun [27] investigated the axial vibration of non-local rods made of a polymer matrix reinforced with SFs under a magnetic field and elastic medium, using the finite element method. Lastly, Kadıoğlu and Yaylı [28] addressed the longitudinal dynamic response of FG viscoelastic nano-beams with flexible supports under the scope of nonlocal elasticity theory.

Recent studies on FG nanostructures have provided significant insights into their mechanical behavior. Alghanmi [29] utilized the nonlocal strain-gradient theory to explore the bends of sandwich nanoplates with FG porous cores and piezomagnetic faces in a hygrothermal environment, highlighting the impact of environmental factors on structural response. In another study, Alghanmi [30] investigated the size-dependent static action of FG nanobeams connected to piezoelectric fiber-reinforced composite actuators, incorporating nonlocal strain-gradient effects to capture nanoscale behavior.

As evidenced by the above literature review, remarkable progress has been achieved in modeling the thermo-mechanical behavior of FG nanobeams using various size-dependent continuum theories. However, despite these advances, no previous study has developed a higher-order shear deformation formulation incorporating an enhanced displacement field with integral unknowns, together with a novel hyperbolic thickness-dependent function for the free vibration analysis of temperature-dependent FG nanobeams within Eringen’s nonlocal elasticity framework. Existing formulations generally rely on classical higher-order beam theories or conventional shear deformation models, which do not simultaneously combine accurate transverse shear representation, satisfaction of traction-free boundary conditions, elimination of shear correction factors, and computational efficiency. These limitations motivate the development of the present formulation.

This study presents a refined analytical framework aimed at accurately capturing the dynamic behavior of advanced nanostructures beneath combined thermal loading and nonlocal effects. The developed model provides a robust theoretical foundation for the efficient integration of these nanoscale elements into next-generation engineering systems and high-performance industrial technologies. The numerical results reported here can be interpreted as valuable reference benchmarks for the construction and development of nanoelectronic devices, nanodrives, nano-oscillators, and nanosensors in which nanobeams play an important structural role.

The main novelty of the present work lies in the development of a refined higher-order shear deformation theory incorporating an enhanced displacement field with integral unknowns and a novel hyperbolic thickness-dependent function proposed by the authors. Unlike conventional higher-order theories, the proposed formulation accurately reproduces the transverse shear strain distribution, inherently satisfies the zero transverse shear stress conditions at the beam surfaces, and eliminates the need for shear correction factors while maintaining a simple analytical framework suitable for nonlocal thermoelastic vibration analysis of FG nanobeams.

## 2. Theoretical Framework

### 2.1. Nonlocal Power-Low FG Nanobeam Model

**Figure 1:**
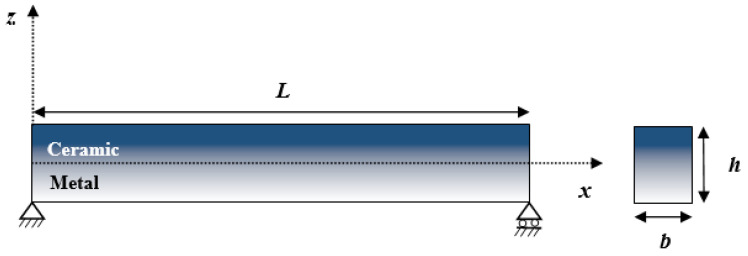
Geometry and coordinates of functionally graded nanobeam.

In the present research, a rectangular FG nanobeam, having geometric dimensions represented by its length (L), thickness (h), and width (b). The beam’s upper and lower surfaces are situated at z=±h/2. The coordinate x is taken along the longitudinal axis, while z represents the thickness direction, as shown in Figure 1. The functional characteristics of the examined FG nanobeam, including Poisson’s ratio (ν), coefficient of thermal expansion (α), Young’s modulus (E), thermal conductivity (κ), and mass density (ρ), will be calculated assuming the following basic power-law pattern [31]:

(1) $$P\left(z\right)=P_{2}+\left(P_{1}-P_{2}\right){\left(\frac{2z+h}{2h}\right)}^{k}$$

The nonlinear variation in the thermoelastic material features of the FG nanobeam will be outlined as follows [32]:

(2) $$P(T)=P_{0}(P_{-1}T^{-1}+1+P_{1}T^{1}+P_{2}T^{2}+P_{3}T^{3})$$

in which T is the environmental temperature; P(T) denotes a generic material property; $P_{-1}$, $P_{0}$, $P_{1}$, $P_{2}$ and $P_{3}$ are the thermally dependent material values specific to each basic material, as indicated in Table 1.

Where $P_{0}$ represents the reference value of the material property, whereas $P_{1}$, $P_{2}$ and $P_{3}$ denote the first-, second-, and third-order temperature-dependent coefficients, respectively. The coefficient $P_{-1}$ corresponds to the inverse-temperature term in the general polynomial expression. Although $P_{-1}$ is equal to zero for the constituent materials considered in this study, it is retained in the formulation to preserve the generality of the temperature-dependent material model, allowing its direct application to other material systems. The units of these coefficients depend on the physical property under consideration so that Equation (2) remains dimensionally consistent.

### 2.2. Review of Nonlocal Elasticity Theory

Traditional elasticity theories are size-independent and can be utilized in structures across various lengths and time scales. However, their accuracy diminishes when the inner characteristic dimension approaches its exterior dimensions, limiting their capability to represent small-scale effects. To overcome this limitation, nonlocal elasticity theories, introduced by Eringen [11,12], incorporate size-dependent continuum mechanics by embedding small-scale impacts directly into the fundamental equations. In contrast to traditional theories, which base stress at a specific location exclusively on local strain, nonlocal elasticity considers the influence of strains at all points within the body on the stress at the point of reference. Eringen’s nonlocal continuum theory [12] expresses the nonlocal stress tensor $\sigma_{ij}$ at a point as

(3) $$\left(1-\mu \nabla^{2}\right)\sigma_{ij}=\sigma_{ij}^{L}$$

where $\nabla^{2}$ denote the Laplacian operator; $\sigma_{ij}^{L}$ is the standard stress tensor at the point associated with the strain by Hooke’s law; and $\mu ={\left(e_{0}a\right)}^{2}$ is the nonlocal parameter that contains the small-scale influence, a represents the inner characteristic length, and $e_{0}$ is a constant specific to each material.

### 2.3. Kinematic Relations

Houari and his colleagues [33,34,35] explored the buckling, bending, and vibration responses of FG-improved composite plates, adopting a novel five-variable quasi-three-dimensional refined plate theory, instead of six in earlier shear deformation models. This article introduces a novel nonlocal parabolic beam theory. By adopting the thick plate theory and neglecting both the transverse normal deformation (i.e., the thickness stretching effect) and deformation along the *y*-direction, the fundamental assumptions for the beam’s displacement field are stated as follows:

(4) $$\begin{array}{c} u_{1}=u_{0}\left(x,t\right)-z\frac{\partial w_{0}}{\partial x}+\gamma_{1}\;\Psi \left(z\right)\int \theta \left(x,t\right)dx\\ u_{3}=w_{0}(x,t) \end{array}$$

The coefficient $\gamma_{1}$ depends on the geometry. Where $u_{i}$, $i=\left(1,3\right)$ are functions of (x,z,t) and $u_{0}$, $w_{0}$, θ are functions of (x,t). The assumed novel theory of the current investigation has the following hyperbolic function.

The proposed hyperbolic thickness-dependent function was specifically designed to accurately represent the transverse shear strain distribution through the beam thickness while inherently satisfying the traction-free boundary conditions at the upper and lower surfaces. Consequently, the proposed refined higher-order shear deformation theory eliminates the need for shear correction factors without increasing the number of unknown displacement variables.

(5) $$\Psi (z)=\frac{3\pi }{2}\left(\frac{\cosh{\left(1/2\right)}^{2}-1}{\cosh{\left(1/2\right)}^{2}}\right)-\frac{\pi \;z}{2}\left(\frac{z^{2}}{2\;h^{2}}\right)$$

The function Ψ(z) represents the proposed hyperbolic transverse shear function, whereas $\Psi^{\prime }\left(z\right)$ are auxiliary functions derived from Ψ(z) and employed in the displacement field and strain expressions. The proposed hyperbolic function has been specifically developed to ensure a realistic distribution of transverse shear strains through the beam thickness while satisfying the zero transverse shear stress conditions at the upper and lower surfaces. Consequently, no shear correction factor is required in the present formulation.

According to Equation (4), the nonzero strain expressions corresponding to the displacement field are given as

(6) $$\varepsilon_{x}=\varepsilon_{x}^{0}+z\;k_{x}^{b}+\Psi \left(z\right)k_{x}^{s}$$

(7) $$\gamma_{xz}=\Psi^{\prime }\left(z\right)\gamma_{xz}^{0}$$

where

(8) $$\varepsilon_{x}^{0}=\frac{\partial u_{0}}{\partial x},\;k_{x}^{b}=-\frac{\partial^{2}w_{0}}{\partial x^{2}},\;k_{x}^{s}=\gamma_{1}\theta ,\;\gamma_{xz}^{0}=\gamma_{1}\int \theta \;dx$$

It should be noted that the adopted strain-displacement relations are linear and do not include geometric nonlinear terms. Therefore, the developed formulation is applicable to the prebuckling regime only.

Equation (6) demonstrates that the transverse shear strain $\gamma_{xz}$ is zero at the nanobeam’s upper and lower surfaces. A shear correction factor is therefore not necessary. The integrals implemented in the preceding equations need to be evaluated through a Navier-type technique and will be represented as follows [33,34,35]:

(9) $$\int \theta \;dx=A^{\prime }\frac{\partial \theta }{\partial x}$$

The coefficient $A^{\prime }$ are determined based on the kind of solution implemented, in this instance, the Navier technique. Hence, $A^{\prime }$ and $\gamma_{1}$ can be written as follows:

(10) $$A^{\prime }=-\frac{1}{\alpha^{2}},\;\gamma_{1}=\alpha^{2}$$

where α is defined in Equation (34).

### 2.4. Constitutive Relations

The nonlocal constitutive equation given in Equation (3) has recently been applied to the analysis of micro- and nanostructural elements. According to this equation, the two-dimensional nonlocal constitutive formulas for an elastic FG nanobeam could be given as follows:

(11) $$\left\{\begin{array}{c} \sigma_{x}\\ \tau_{xz} \end{array}\right\}\left(1-\mu \;\nabla^{2}\right)=\left[\begin{array}{cc} Q_{11} & 0\\ 0 & Q_{55} \end{array}\right]\left\{\begin{array}{c} \varepsilon_{x}\\ \gamma_{xz} \end{array}\right\}$$

where $\left(\sigma_{x},\tau_{xz}\right)$ and $\left(\varepsilon_{x},\gamma_{xz}\right)$ are stress and strain components, accordingly. $\mu ={\left(e_{0}a\right)}^{2}$ and $Q_{ij}$ are the stiffness variables and are defined as

(12) $$Q_{11}=E\left(z\right),\;Q_{55}=G\left(z\right).$$

(13) $$G(z)=(G_{c}-G_{m}){\left(\frac{2z+h}{2h}\right)}^{k}+G_{m}$$

It should be noted that the functionally graded material is assumed to remain locally isotropic at each point through the thickness. Therefore, despite the continuous variation in material properties, no constitutive coupling between normal and transverse shear strains is considered. Furthermore, the adopted hyperbolic shear-deformation function guarantees the vanishing of transverse shear strains and stresses at the upper and lower beam surfaces, ensuring compatibility with the traction-free boundary conditions within the nonlocal elasticity framework.

### 2.5. Equations of Motion

Following Hamilton’s concept, one can construct the motion equations that explain the free vibration action of FG nanobeams [36]

(14) $$0=\int_{0}^{t}\delta (U+V-K)dt$$

at which t is the time. U is strain energy, V is work performed by acting forces, and  K is the variation in motion energy of the beam. The beam’s variation in strain energy is described as

(15) $$\delta \;U=\int_{V}\left(\sigma_{x}\delta \;\varepsilon_{x}+\tau_{xz}\delta \;\gamma_{xz}\right)\;dA\;dz=\int_{A}\left\{N_{x}\delta \;\varepsilon_{x}^{0}+M_{x}^{b}\delta \;k_{x}^{b}+M_{x}^{s}\delta \;k_{x}^{s}+S_{xz}^{s}\delta \;\gamma_{xz}^{0}\right\}\;\;dA$$

in which the stress resultants $N_{x}$, $M_{x}^{b}$, $M_{x}^{s}$ and $S_{xz}^{s}$ are described by

(16) $$\left(N_{x},M_{x}^{b},M_{x}^{s}\right)=\int_{-h/2}^{h/2}\left(1,z,\Psi (z)\right)\sigma_{x}dz$$

(17) $$\left(S_{xz}^{s}\right)=\int_{-h/2}^{h/2}\Psi^{\prime }(z)\left(\tau_{xz}\right)$$

The virtual variation in the work performed by forces employed may be stated in the form

(18) $$\delta \;V=\int_{0}^{L}\left[\left(N^{T}\right)\;\left(\frac{\partial w}{\partial x}\frac{\partial \delta \;w}{\partial x}\right)-k_{w}w\delta \;w+k_{s}\left(\frac{\partial w}{\partial x}\frac{\partial \delta \;w}{\partial x}\right)\right]dx$$

in which $k_{w}$ and $k_{s}$ are the linear and shear coefficients of the elastic base. $N^{T}$ are the forces employed owing to changes in temperature and states as

(19) $$N^{T}=\int_{-h/2}^{h/2}E(z)\alpha (z,T)(T-T_{0})dz$$

in which $T_{0}$ is the standard temperature. The variation in motion energy is written as

(20) $$\begin{array}{l} \delta \;K=\int_{V}\left[\dot{u}\;\delta \;\dot{u}+\dot{w}\;\delta \;\dot{w}\right]\;\;\rho (z)\;dV\\ =\int_{A}\left\{I_{0}\left({\dot{u}}_{0}\delta {\dot{u}}_{0}+{\dot{w}}_{0}\delta {\dot{w}}_{0}\right)\right.-I_{1}\left({\dot{u}}_{0}\frac{\partial \delta {\dot{w}}_{0}}{\partial x}+\frac{\partial {\dot{w}}_{0}}{\partial x}\delta {\dot{u}}_{0}\right)\\ +\;J_{1}\left(\left(\gamma_{1}\;A^{\prime }\right)\;\left({\dot{u}}_{0}\frac{\partial \delta \dot{\theta }}{\partial x}+\frac{\partial \dot{\theta }}{\partial x}\delta {\dot{u}}_{0}\right)\right)+I_{2}\left(\frac{\partial {\dot{w}}_{0}}{\partial x}\frac{\partial \delta \;{\dot{w}}_{0}}{\partial x}\right)+K_{2}\left({\left(\gamma_{1}A^{\prime }\right)}^{2}\left(\frac{\partial \dot{\theta }}{\partial x}\frac{\partial \delta \;\dot{\theta }}{\partial x}\right)\right)\\ \left.-J_{2}\left(\left(\gamma_{1}A^{\prime }\right)\left(\frac{\partial {\dot{w}}_{0}}{\partial x}\frac{\partial \delta \;\dot{\theta }}{\partial x}+\frac{\partial \dot{\theta }}{\partial x}\frac{\partial \delta \;{\dot{w}}_{0}}{\partial x}\right)\right)\right\}dA \end{array}$$

where the superposed dot indicates differentiation with respect to t; ρ(z) denotes the mass density, and the mass inertia terms $\left(I_{0},I_{1},I_{2},J_{1},J_{2},K_{2}\right)$ are defined as follows:

(21) $$\left(I_{0},I_{1},I_{2}\right)=\int_{-h/2}^{h/2}\left(1,z,z^{2}\right)\;\;\rho (z)dz$$

(22) $$\left(J_{1},J_{2},K_{2}\right)=\int_{-h/2}^{h/2}\left(\Psi (z),z\;\Psi (z),\Psi^{2}(z)\right)\;\;\;\rho (z)dz$$

by incorporating the formulas for δ U and δ K from Equations (15) and (20) into Equation (14), integratingby parts, and summing the coefficients of $\delta \;u_{0}$, $\delta \;w_{0}$ and δ θ, the next equations of motion of the beam are generated.

Applying integration by parts to the virtual work expression yields the governing equations together with the corresponding boundary terms, from which the essential and natural boundary conditions are derived.

(23) $$\delta \;u_{0}:\frac{\partial N_{x}}{\partial x}=I_{0}{\ddot{u}}_{0}-I_{1}\frac{\partial {\ddot{w}}_{0}}{\partial x}+\gamma_{1}A^{\prime }\;J_{1}\frac{\partial \ddot{\theta }}{\partial x}$$

(24) $$\delta \;w_{0}:\frac{\partial^{2}M_{x}^{b}}{\partial x^{2}}+\left(N^{T}\right)\frac{\partial^{2}w}{\partial x^{2}}+k_{w}w-k_{s}\frac{\partial^{2}w}{\partial x^{2}}=I_{0}{\ddot{w}}_{0}+I_{1}\left(\frac{\partial {\ddot{u}}_{0}}{\partial x}\right)-I_{2}\frac{\partial^{2}{\ddot{w}}_{0}}{\partial x^{2}}+J_{2}\;\left(\gamma_{1}\;A^{\prime }\frac{\partial^{2}\ddot{\theta }}{\partial x^{2}}\right)\;$$

(25) $$\delta \;\theta :\gamma_{1}\;M_{x}^{s}-\gamma_{1}\;A\prime \frac{\partial S_{xz}^{s}}{\partial x}=\;J_{1}\;\left(\gamma_{1}\;A^{\prime }\frac{\partial {\ddot{u}}_{0}}{\partial x}\right)+K_{2}\left({\left(\gamma_{1}\;A^{\prime }\right)}^{2}\;\;\frac{\partial^{2}\ddot{\theta }}{\partial x^{2}}\right)-J_{2}\;\left(\gamma_{1}\;A^{\prime }\frac{\partial^{2}{\ddot{w}}_{0}}{\partial x^{2}}\right)$$

It should be noted that the governing equations are obtained by applying integration by parts to the virtual work expression. The corresponding boundary terms lead naturally to the essential and natural boundary conditions of the beam.

The integration by parts performed in the variational formulation leads naturally to the essential and natural boundary conditions associated with the proposed beam model. For the numerical examples considered in the present study, simply supported (S–S) boundary conditions are imposed at both beam ends (x=0 and x=L). The corresponding boundary conditions are $w_{0}=0$, Θ=0, $M_{x}^{b}=0$, $M_{x}^{s}=0$, where $\Theta \left(x,t\right)=\int \theta \left(x,t\right)dx$ is treated as a generalized displacement variable associated with the transverse shear deformation. These boundary conditions prevent transverse displacement at the supports while allowing free rotations, thereby representing the classical simply supported beam configuration. They are fully compatible with the Navier solution procedure adopted in the present work.

Combining Equations (8) and (11) into Equations (16) and (17) and integrating across the thickness of the beam, the stress resultis linked to the generalized displacements ($u_{0}$, $w_{0}$, θ) through the equations:

(26) $$\left\{\begin{array}{c} N_{x}\\ M_{x}^{b}\\ M_{x}^{b} \end{array}\right\}-\mu \frac{\partial^{2}}{\partial x^{2}}\left\{\begin{array}{c} N_{x}\\ M_{x}^{b}\\ M_{x}^{b} \end{array}\right\}=\left[\begin{array}{ccc} A_{11} & B_{11} & \;B_{11}^{s}\\ B_{11} & D_{11} & \;D_{11}^{s}\\ \;B_{11}^{s} & \;D_{11}^{s} & \;H_{11}^{s} \end{array}\right]\left\{\begin{array}{c} \varepsilon_{x}^{0}\\ k_{x}^{b}\\ k_{x}^{s} \end{array}\right\}$$

(27) $$S_{xz}^{s}-\mu \frac{\partial^{2}S_{xz}^{s}}{\partial x^{2}}=A_{55}^{s}\;\gamma_{xz}^{0}$$

where

(28) $$\left(A_{11},A_{11}^{s},B_{11},D_{11},B_{11}^{s},D_{11}^{s},H_{11}^{s}\right)=\int_{-h/2}^{h/2}Q_{ij}\left(1,g^{2}(z),z,z^{2},\Psi (z),z\;\Psi (z),\Psi^{2}(z)\right)\;dz$$

Substituting from Equations (26) and (27) into Equations (23), (24) and (25), the equilibrium equations could be written in regard to displacements ($\;u_{0}$, $w_{0}$ and θ) as

(29) $$A_{11}d_{11}u_{0}-B_{11}d_{111}w_{0}+B_{11}^{s}\gamma_{1}\;\;d_{1}\theta =\left(1-\mu \nabla^{2}\right)\;\left[I_{0}{\ddot{u}}_{0}-I_{1}\;d_{1}{\ddot{w}}_{0}+J_{1}\;A^{\prime }\gamma_{1}d_{1}\ddot{\theta }\right],$$

(30) $$B_{11}\;d_{111}u_{0}-D_{11}d_{1111}w_{0}+D_{11}^{s}\;\gamma_{1}\;d_{11}\theta \;=\left(1-\mu \nabla^{2}\right)\;\left(I_{0}{\ddot{w}}_{0}+I_{1}\;\left(d_{1}{\ddot{u}}_{0}\right)\;-I_{2}\;d_{11}{\ddot{w}}_{0}+J_{2}\;\left(\gamma_{1}A^{\prime }\;\;d_{11}\ddot{\theta }\right)\right)\;$$

(31) $$\begin{array}{l} -B_{11}^{s}k_{1}\;d_{1}u_{0}D_{11}^{s}\gamma_{1}\;d_{11}w_{0}-H_{11}^{s}\;\gamma_{1}^{2}\;\;\theta +A_{55}^{s}\;\;{\left(k_{1}\;A^{\prime }\right)}^{2}\;d_{11}\theta +D_{11}^{s}\gamma_{1}\;d_{11}w_{0}-H_{11}^{s}\;\gamma_{1}^{2}\;\;\theta +A_{55}^{s}\;\;{\left(\gamma_{1}\;A^{\prime }\right)}^{2}\;d_{11}\theta \\ =\left(1-\mu \nabla^{2}\right)\;\left[-\;J_{1}\left(\gamma_{1}\;A^{\prime }d_{1}{\ddot{u}}_{0}\right)+J_{2}\left(\gamma_{1}\;A^{\prime }d_{11}{\ddot{w}}_{0}\right)-K_{2}\left({\left(\gamma_{1}A^{\prime }\right)}^{2}\;d_{11}\ddot{\theta }\right)\;\right] \end{array}$$

in which the differential operators $d_{ij}$, $d_{ijl}$ and $d_{ijlm}$ are as follows:

(32) $$d_{ij}=\frac{\partial^{2}}{\partial x_{i}\partial x_{j}}\;d_{ijl}=\frac{\partial^{3}}{\partial x_{i}\partial x_{j}\partial x_{l}},\;d_{ijlm}=\frac{\partial^{4}}{\partial x_{i}\partial x_{j}\partial x_{l}\partial x_{m}},\;d_{i}=\frac{\partial }{\partial x_{i}},\;(i,j,l,m=1,2).$$

## 3. Exact Solution for FG Nanobeam

In this study, we focus on obtaining the exact solutions of Equations (29)–(31) for a simply supported FG nanobeam. By applying the Navier solution method, the displacements ($u_{0}$, $w_{0}$ and θ) are expressed as follows:

(33) $$\left\{\begin{array}{c} u_{0}\\ w_{0}\\ \theta \end{array}\right\}=\left\{\begin{array}{c} U_{mn}\;e^{i\omega \;t}\cos(\alpha \;x)\\ W_{mn}\;e^{i\omega \;t}\sin(\alpha \;x)\\ X_{mn}\;e^{i\omega \;t}\sin(\alpha \;x) \end{array}\right\}$$

(34) α=mπ/L

where $i=\sqrt{-1}$, α=mπ/L, ω is the natural frequency, and ($U_{mn}$, $W_{mn}$, $X_{mn}$) represent the unknown maximum displacement values.Inserting Equation (33) into Equations (29)–(31) yields the analytical solutions:

(35) $$\left(\left[\begin{array}{ccc} a_{11} & a_{12} & a_{13}\\ a_{12} & a_{22} & a_{23}\\ a_{13} & a_{23} & a_{33} \end{array}\right]-\lambda \;\omega^{2}\left[\begin{array}{ccc} M_{11} & M_{12} & M_{13}\\ M_{12} & M_{22} & M_{23}\\ M_{13} & M_{23} & M_{33} \end{array}\right]\right)\left\{\begin{array}{c} U_{mn}\\ W_{mn}\\ X_{mn} \end{array}\right\}=\left\{\begin{array}{c} 0\\ 0\\ 0 \end{array}\right\}$$

where

(36) $$\begin{array}{l} a_{11}=-A_{11}\alpha^{2},\;a_{12}=B_{11}\alpha^{3},\;\;a_{13}=\alpha \left(\gamma_{1}B_{11}^{s}\right),\;\;\\ a_{22}=-\;D_{11}\alpha^{4},\;\;a_{23}=-k_{1}\;\;\left(D_{11}^{s}\alpha^{2}\right),\\ a_{33}=-k_{1}\left(H_{11}^{s}\gamma_{1}\right)-{\left(\gamma_{1}A^{\prime }\right)}^{2}A_{55}^{s}\alpha^{2}\\ M_{11}=-I_{0},\;\;M_{12}=\alpha I_{1},\;\;M_{13}=-\gamma_{1}\;J_{1}A^{\prime }\;\alpha ,\;\;\\ M_{22}=-I_{0}-I_{2}(\alpha^{2}),\;\;M_{23}=\;J_{2}\left(\gamma_{1}A^{\prime }\alpha^{2}\right),\;\;\;\\ M_{33}=-\;K_{2}\;\left({\left(\gamma_{1}A^{\prime }\right)}^{2}\alpha^{2}\right),\;\;\;\;\lambda =1+\mu \left(\alpha^{2}\right) \end{array}$$

## 4. Various Thermal Environments

### 4.1. Uniform Temperature Rise (UTR)

The FG nanobeam at standard temperature $T_{0}$, is uniformly elevated to a final value T, where the temperature changes are $\Delta T=T-T_{0}$.

### 4.2. Linear Temperature Rise (LTR)

The second model considers the distribution of linear temperature within the entire FG nanobeam’s thickness, where the final temperature T is determined in the following manner [37].

(37) $$T=T_{m}+\Delta T\;\left(\frac{z}{h}+\frac{1}{2}\right)$$

### 4.3. Nonlinear Temperature Rise (NLTR)

As the thickness of the FG nanobeam rises in the third case, the temperature increases nonlinearly. The temperature field is calculated by solving the following one-dimensional steady-state heat transfer equation [37]:

(38) $$\begin{array}{l} -\frac{d}{dz}\left(\kappa \left(z,T\right)\frac{dT}{dz}\right)=0\\ T\left(\frac{h}{2}\right)=T_{c},\;T\left(-\frac{h}{2}\right)=T_{m} \end{array}$$

The following expression can be obtained by solving the aforementioned equation (Equation (38)), according to the boundary conditions $T\left(h/2\right)=T_{c}$ and $T\left(-h/2\right)=T_{m}$ at the upper and lower free surfaces of the FG nanobeam:

(39) $$T=T_{m}+\left(\Delta T\right)\frac{\int_{-h/2}^{z}\frac{1}{\kappa \left(z,T\right)}dz}{\int_{-h/2}^{h/2}\frac{1}{\kappa \left(z,T\right)}dz}$$

in which $\Delta T=T-T_{0}$ denotes variation in temperature.

Where $T_{0}$ denotes the reference (initial) temperature, T is the temperature at an arbitrary point through the beam thickness, $T_{c}$ and $T_{m}$ represent the temperatures at the ceramic-rich and metal-rich surfaces, respectively, and $\Delta T=T-T_{0}$ is the temperature rise. For the present FG nanobeam, the upper surface $\left(z=\frac{h}{2}\right)$ is ceramic-rich, whereas the lower surface $\left(z=-\frac{h}{2}\right)$ is metal-rich.

For clarity, the above temperature notations are used consistently throughout the manuscript for the uniform (UTR), linear (LTR), and nonlinear (NLTR) thermal loading cases.

## 5. Discussion of the Results

This section presents a detailed analysis of the nondimensional natural frequencies of a simply supported FG nanobeam under various temperature (uniform, linear, and nonlinear) distributions. The results are obtained using a new higher-order shear deformation theory founded on an improved displacement field with integral unknowns and a hyperbolic thickness-dependent function. This formulation accurately accounts for transverse shear strains and does not require a shear correction factor. The accuracy of the proposed results is validated by comparing them to published solutions in the literature.

In this study, the FG nanobeam is composed of two constituent materials, $Si_{3}N_{4}$ and SUS304, whose characteristics change with thickness depending on a power law distribution. The metallic surface temperature $T_{m}$ of the simply supported P-FG nanobeam is assumed to rise to the reference temperature $T_{0}$, with a temperature difference of $T_{m}-T_{0}=5\;K$. For clarity and ease of comparison, the natural frequencies are reported in the following dimensionless form [37,38]:

(40) $$\hat{\omega }=\omega L^{2}\sqrt{\frac{\rho_{c}A}{E_{c}I}},\;I=\frac{bh^{3}}{12}$$

Table 2 shows a comparison of the nondimensional natural frequencies $\hat{\omega }$ of a simply supported FG nanobeam. The results are estimated for a variety of nonlocal parameters (μ = 0, 1, 2, 3, and 4) and material gradient indices (k = 0, 0.2, 1, and 5). The results are compared to those predicted by the Timoshenko beam model [37,38], as well as those reported by Bendaida et al. [39], who used an advanced cubic shear deformation formulation with only two unknown variables.

As shown in Table 2, the proposed formulation is highly consistent with the reference solutions developed using Timoshenko beam theory [37,38], as well as the cubic shear deformation approach presented by Bendaida et al. [39]. This consistency is maintained across all considered nonlocal parameters μ and material gradient indices k, confirming the model’s reliability for small-scale FG nanobeam analysis.

Table 3, Table 4 and Table 5 list the fundamental dimensionless natural frequencies of a simply supported FG nanobeam under uniform, linear, and nonlinear temperature distributions, respectively. The frequencies are calculated for temperature rises of ΔT= 10, 30, 60 K, nonlocal parameters μ = 0, 1, 2, 3, 4, and material gradient indices k = 0, 0.2, 1, 5. The findings demonstrate that the dimensionless frequency decreases with increasing nonlocal parameter, material gradient index, and temperature change. The completely ceramic beam (k = 0) also exhibits the highest natural frequencies due to its greater stiffness and enhanced resistance to thermal effects.

The reduction in the dimensionless natural frequencies with increasing temperature is primarily attributed to the degradation of the temperature-dependent elastic properties of the constituent materials. Since the present model is based on linear kinematics, geometric stiffening effects associated with post-buckling deformation are not considered. Therefore, the present analysis is restricted to the prebuckling regime. It is also observed that the nonlocal parameter significantly influences the thermal stability of the FG nanobeam. Increasing the nonlocal parameter enhances the size-dependent softening effect, which reduces the effective structural stiffness and leads to lower natural frequencies. Consequently, the critical thermal buckling temperature decreases as the nonlocal parameter increases, indicating that nonlocality advances the onset of thermal instability. Physically, the nonlocal softening effect and the thermally induced compressive stresses act simultaneously to reduce the stability of the nanobeam.

**Figure 2:**
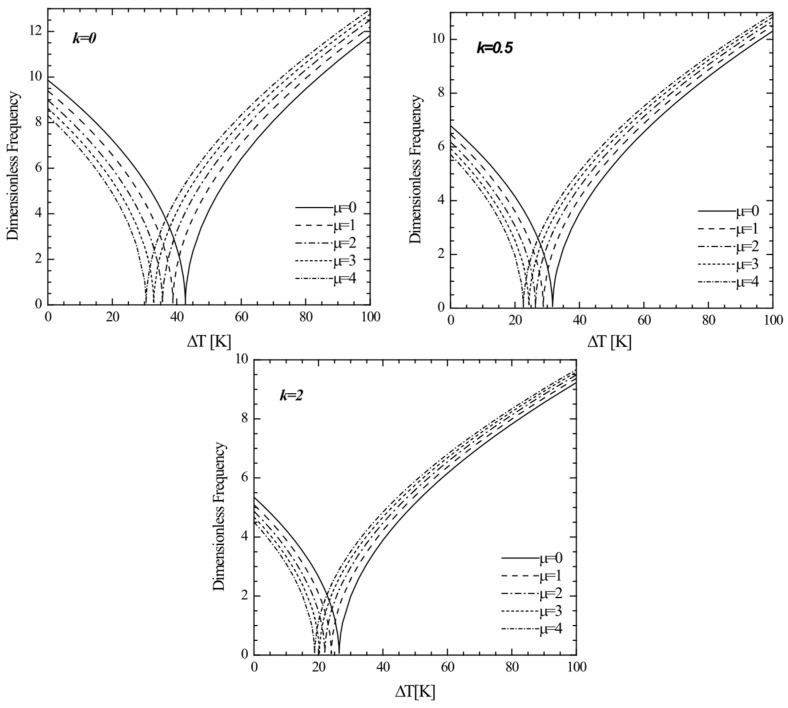
Variations in the first dimensionless natural frequency $\hat{\omega }$ of the FG nanobeam with respect to temperature change ΔT for different values of the nonlocal parameter μ, under uniform thermal loading (UTR), with L/h=50 and k=0.

**Figure 3:**
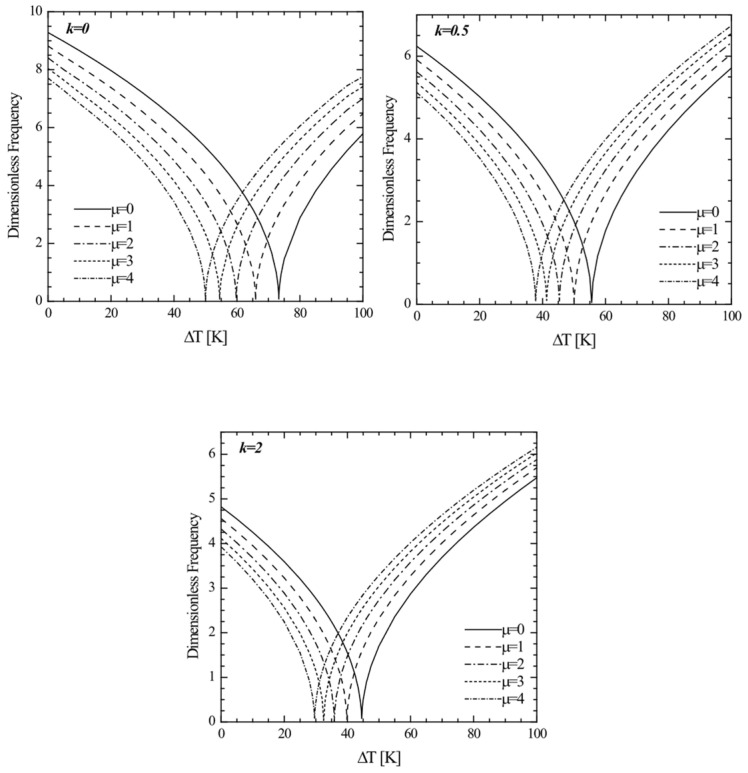
Variations in the first dimensionless natural frequency $\hat{\omega }$ of the FG nanobeam with respect to temperature change ΔT for different values of the nonlocal parameter μ, under linear thermal loading (LTR), with L/h=50 and k=0.5.

**Figure 4:**
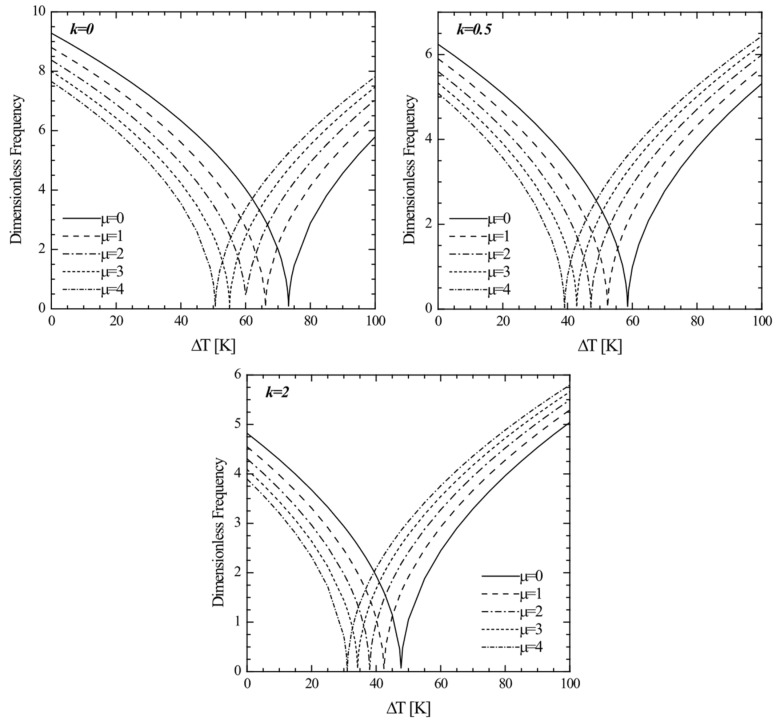
Variations in the first dimensionless natural frequency $\hat{\omega }$ of the FG nanobeam with respect to temperature change ΔT for different values of the nonlocal parameter μ, under nonlinear thermal loading (NLTR), with L/h=50 and k=2.

Figure 2, Figure 3 and Figure 4 describe how the initial dimensionless natural frequency $\hat{\omega }$ of a simply supported FG nanobeam (with L/h=50) varies with the nonlocal parameter μ and the material gradient index k under uniform (UTR), linear (LTR), and nonlinear (NLTR) thermal loadings. The results suggest that the dimensionless fundamental frequency decreases as the temperature variation increases and eventually reaches zero. This state corresponds to the critical thermal buckling temperature of the FG nanobeam, indicating the onset of instability. Since the present formulation is based on linear strain-displacement relations, the analysis is restricted to the prebuckling regime, and no conclusions are drawn regarding the post-buckling behavior. In addition, larger values of the material gradient index k and the nonlocal parameter μ reduce the fundamental frequency. After exceeding the critical temperature, however, the opposite trend is observed, where the frequency increases as k and μ become higher.

The highest dimensionless natural frequencies $\hat{\omega }$ are obtained for nanobeams exposed to a uniform temperature rise (UTR) with k = 0, corresponding to the stiffest configuration compared to beams with higher material gradient indices.

The decrease in the nondimensional natural frequency with increasing temperature is attributed to the progressive degradation of the effective material stiffness and the development of thermally induced compressive stresses, which reduce the structural rigidity of the FG nanobeam.

**Figure 5:**
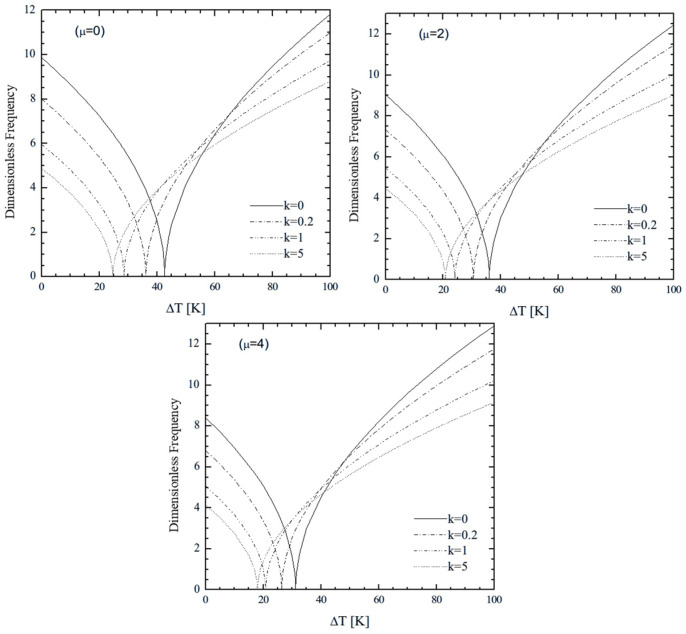
Variations in the first dimensionless natural frequency $\hat{\omega }$ of the FG nanobeam with respect to temperature change ΔT for different values of the volume fraction index k and nonlocal parameter μ, under uniform thermal loading.

**Figure 6:**
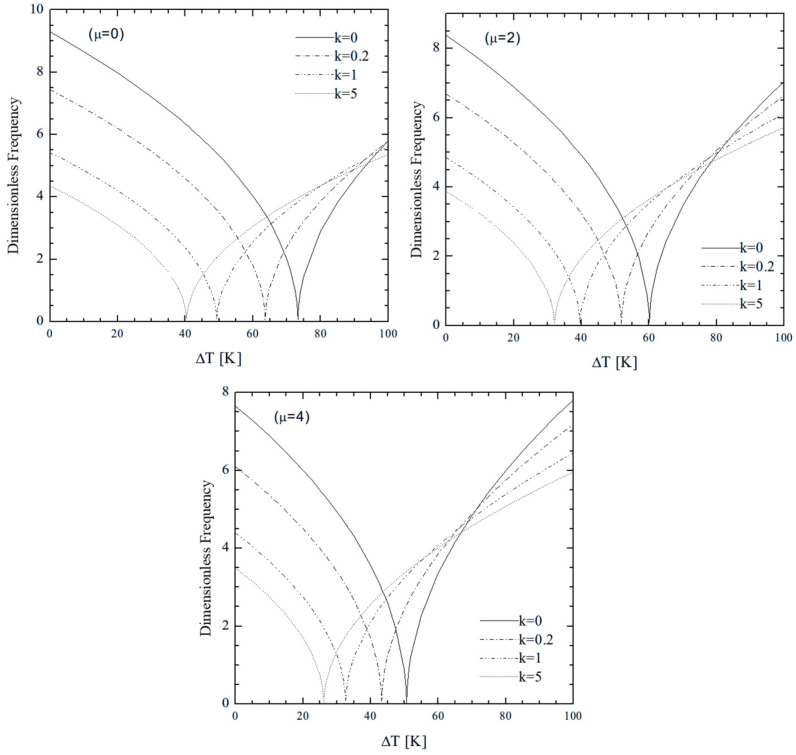
Variations in the first dimensionless natural frequency $\hat{\omega }$ of the FG nanobeam with respect to temperature change ΔT for different values of the volume fraction index k and nonlocal parameter μ, under linear thermal loading.

Figure 5 and Figure 6 present the behavior of the dimensionless fundamental frequency $\hat{\omega }$ for a slender FG nanobeam (L/h=50) in relation to the volume fraction index k, the small-scale parameter μ, and the temperature increase ΔT under linear (LTR) and uniform (UTR) thermal loading conditions. It can be observed that the fundamental frequency decreases steadily as the small-scale parameter μ increases, independent of the type of thermal loading applied.

It is also evident that the gap between the critical temperature values for different material gradient indices (k = 0, 0.2, 1, 5) becomes significantly more pronounced under linear thermal loads, indicating a greater sensitivity to the temperature distribution. Finally, the lowest values of $\hat{\omega }$ are associated with the smallest nonlocality parameters μ, confirming the dominant influence of scale impacts on the vibrational properties of FG nanostructures.

It can be observed that the reduction in the natural frequency is more pronounced under uniform thermal loading than under linear thermal loading. This behavior is attributed to the fact that a uniform temperature rise generates a stronger thermally induced axial compressive force throughout the beam thickness, leading to a larger reduction in the effective stiffness. In contrast, a linear temperature distribution also produces thermal moments due to the through-thickness temperature gradient, which modifies the internal stress distribution and results in a less severe reduction in the structural stiffness.

**Figure 7:**
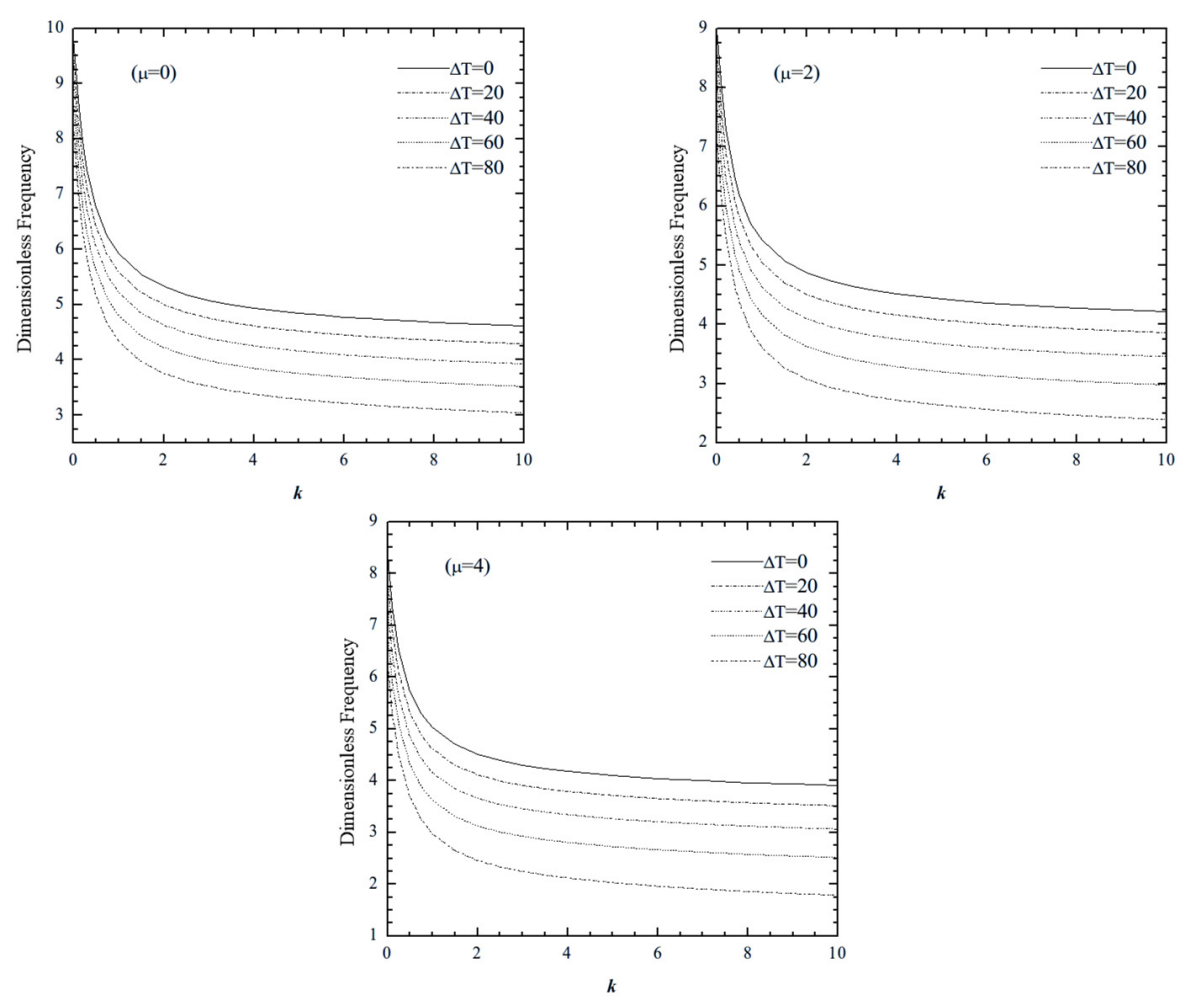
The variation in the first dimensionless frequency $\hat{\omega }$ of the FG nanobeam versus the material graduation k and temperatures for different nonlocality parameters μ, under uniform thermal loading (L/h=20).

**Figure 8:**
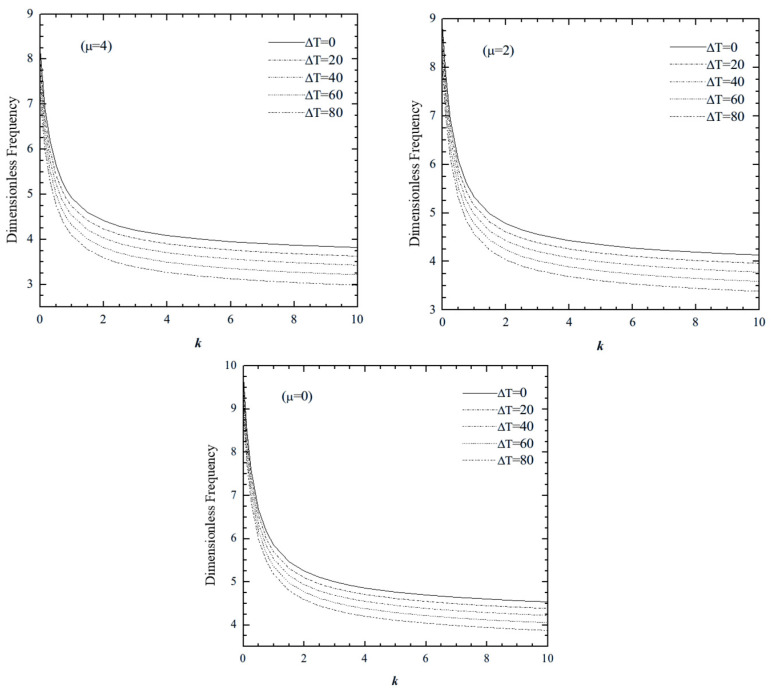
The variation in the first dimensionless frequency $\hat{\omega }$ of the FG nanobeam versus the material graduation k and temperatures for different nonlocality parameters μ, under linear thermal loading (L/h=20).

Figure 7 and Figure 8 illustrate the influence of the temperature increase ΔT and the power-law index k on the dimensionless fundamental frequency $\hat{\omega }$ of a simply supported moderately short FG nanobeam under uniform and linear thermal loading conditions, respectively, for small-scale parameters μ = 0, 2, and 4. The results reveal that increasing either ΔT or k reduces $\hat{\omega }$, as lower values of k lead to greater structural flexibility. Moreover, the frequency differences between various ΔT values are significantly more pronounced under uniform thermal loading.

Increasing the power-law index increases the metallic volume fraction within the beam. Since the metallic constituent possesses a lower Young’s modulus than the ceramic constituent, the overall structural stiffness decreases, which leads to a reduction in the nondimensional natural frequency.

**Figure 9:**
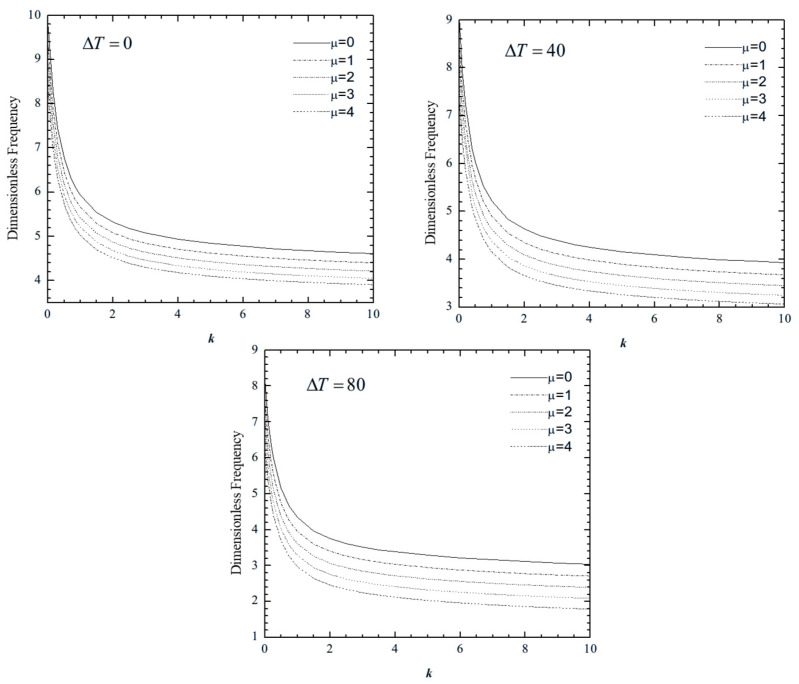
Variation in the first dimensionless frequency $\hat{\omega }$ of the FG nanobeam with respect to the volume fraction index k and the nonlocality parameter μ, for different uniform thermal loading cases (L/h=20).

**Figure 10:**
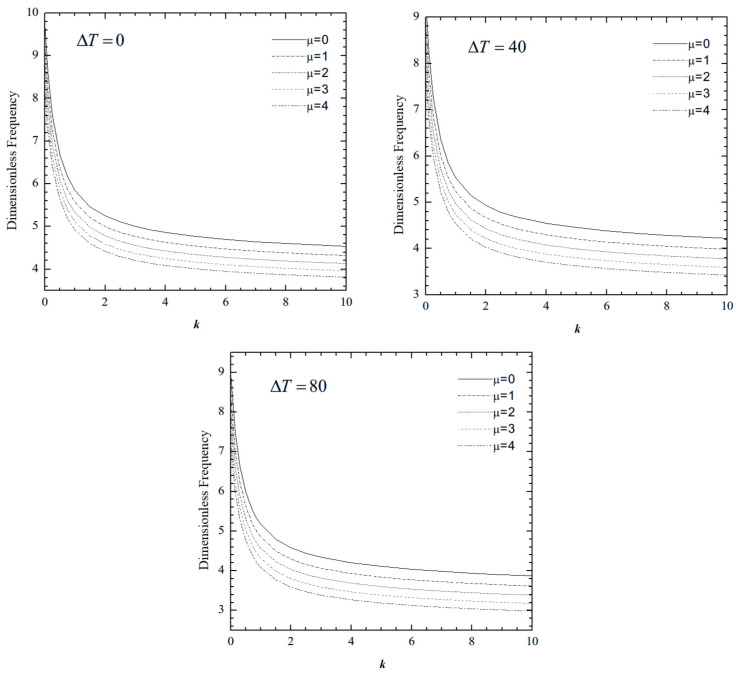
Variation in the first dimensionless frequency $\hat{\omega }$ of the FG nanobeam with respect to the volume fraction index k and the nonlocality parameter μ, for different linear thermal loading cases (L/h=20).

Figure 9 and Figure 10 present the variation in the nondimensional fundamental frequency $\hat{\omega }$ of the FG nanobeam as a function of the nonlocal parameter μ and the material gradient index k under linear and uniform temperature distributions, respectively. The results indicate that increasing either μ or k leads to a reduction in $\hat{\omega }$. Moreover, the plotted curves show that the maximum values of $\hat{\omega }$ occur when the temperature variation is ΔT=0.

**Figure 11:**
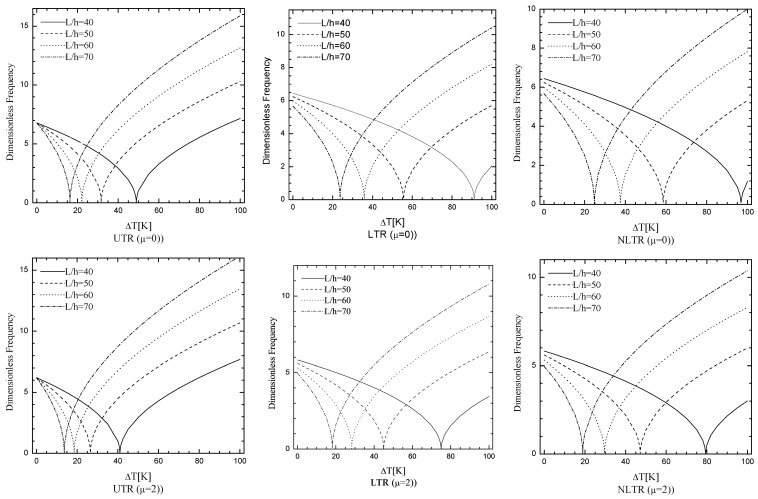

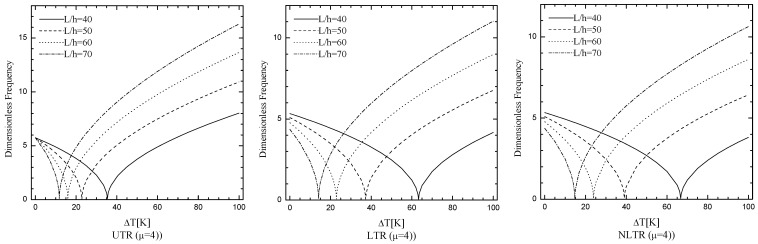
Variations in the first dimensionless natural frequency $\hat{\omega }$ of the FG nanobeam versus the temperature change for different values of nonlocal parameters μ, aspect ratios “L/h” and thermal loading (k=0.5).

Figure 11 demonstrates the influence of the small-scale parameter μ and the slenderness L/h ratio on the nondimensional fundamental frequency $\hat{\omega }$ of a simply supported P-FG nanobeam under linear, nonlinear, and uniform thermal loading conditions. An increase in the aspect ratio L/h reduces the bending stiffness of the beam, resulting in lower natural frequencies, in agreement with classical beam mechanics.

The results indicate that $\hat{\omega }$ decreases as the temperature variation ΔT and the aspect ratio L/h increase at temperatures below the critical value. In addition, the minimum values of $\hat{\omega }$ correspond to the largest nonlocal parameter (μ=4), whereas the maximum frequencies are consistently associated with the FG nanobeam subjected to uniform thermal loading (UTR).

It should be emphasized that the present formulation is based on linear kinematic assumptions and is therefore applicable only to the prebuckling regime. The analysis of post-buckling behavior, including membrane stiffening effects and geometrical nonlinearities, requires a nonlinear theoretical framework and is beyond the scope of the present work. As expected from classical beam mechanics, increasing the aspect ratio reduces the bending stiffness and consequently lowers the natural frequency. The present linear formulation is applicable only up to the critical thermal buckling state, beyond which a nonlinear post-buckling analysis would be required.

It should be emphasized that the present formulation is based on linear strain-displacement relations and is therefore restricted to the prebuckling regime. The post-buckling response is beyond the scope of the present work. The investigation of post-buckling vibrations would require the incorporation of geometric nonlinearities and membrane stiffening effects, which are not considered in the current model.

Overall, the numerical results demonstrate that the vibration behavior of FG nanobeams is governed by the combined effects of material gradation, temperature-dependent material properties, structural stiffness, mass distribution, and nonlocal elasticity. The interplay among these factors determines the overall dynamic response under different thermal environments.

## 6. Conclusions

To effectively simulate the free vibration performance of temperature-dependent FG nanobeams under various thermal environments, an advanced higher-order shear deformation theory has been developed, incorporating integral unknowns and a novel hyperbolic thickness-dependent function. The model, formulated within Eringen’s nonlocal elasticity framework and solved using Navier’s method, eliminates the need for shear correction factors while maintaining high accuracy. Validation against existing analytical solutions confirms its reliability, and a comprehensive parametric study demonstrates the significant influences of thermal loading type, temperature variation, power-law index, nonlocal parameter, and aspect ratio on the fundamental frequencies. The proposed formulation provides an accurate and efficient analytical tool for the vibration analysis of FG nanobeams in thermally affected environments.

Finally, the present study opens several promising research directions. Future developments may incorporate advanced nanoscale foundation models, such as the convolutive elastic substrate media proposed by Devnath and Islam [40], to provide a more realistic representation of the interaction between nanobeams and their supporting environments. Moreover, the recently developed di-convolution nonlocal elasticity framework [41] offers an attractive alternative for modeling size-dependent effects beyond the classical differential Eringen model adopted in the present work. Further investigations may also focus on the stability analysis of functionally graded nanobeams resting on pretensioned membrane foundations using stress-driven nonlocal integral formulations [42]. In addition, the interaction between thermal environments, magnetic fields, and size-dependent effects in graded nanostructures [43] represents another promising extension of the present formulation. It should also be noted that the stress-driven integral nonlocal method proposed by Barretta et al. [44] constitutes a robust alternative to Eringen’s differential nonlocal elasticity theory and may be adopted in future studies to overcome some of the limitations associated with the differential formulation. Such developments would considerably broaden the applicability of the proposed model and contribute to the advancement of more comprehensive theoretical frameworks for nanoscale structural systems.

## Figures and Tables

**Table 1:** Temperature-dependent material properties of FGM constituents [27].

Material	Properties	P_0_	P_−1_	P_1_	P_2_	P_3_
Si_3_N_4_	*E* (Pa)	3.4843 × 10^11^	0	−3.070 × 10^−4^	2.160 × 10^−7^	−8.946 × 10^−11^
	α (K^−1^)	5.8723 × 10^−6^	0	9.095 × 10^−4^	0	0
	*ρ* (kg/m^3^)	2370	0	0	0	0
	*ν*	0.24	0	0	0	0
SUS 304	*E* (Pa)	2.0104 × 10^11^	0	3.079 × 10^−4^	−6.534 × 10^−7^	0
	α (K^−1^)	12.33 × 10^−6^	0	8.086 × 10^−4^	0	0
	*ρ* (kg/m^3^)	8166	0	0	0	0
	*ν*	0.3262	0	−2.002 × 10^−4^	3.797 × 10^−7^	0

**Note:** The coefficients $P_{-1}$, $P_{0}$, $P_{1}$, $P_{2}$ and $P_{3}$ are the temperature-dependent material constants associated with Equation (2). Their units depend on the corresponding material property (Young’s modulus, thermal expansion coefficient, density, or Poisson’s ratio), ensuring dimensional consistency of the adopted polynomial expression.

**Table 2:** Comparison of the nondimensional fundamental frequency $\hat{\omega }$ for a simply supported (S–S) FG nanobeam with various material-gradient indices, for L = 10 nm and h = 0.2 nm.

μ (nm^2^)	Rahmani and Pedram [34]	Ebrahimi and Salari [33]	Bendaida et al. [35]	Present	Rahmani and Pedram [34]	Ebrahimi and Salari [33]	Bendaida et al. [35]	Present
	k=0				k=0.2			
0	9.8631	9.86315733	9.8631573	9.8632	8.6895	8.68954599	8.6895657	8.6895
1	9.4097	9.40973040	9.4097304	9.4097	8.2901	8.29007206	8.2900909	8.2901
2	9.0136	9.01358936	9.0135894	9.0135	7.9411	7.94106762	7.9410857	7.9411
3	8.6636	8.66360601	8.6636060	8.6636	7.6327	7.63272858	7.6327459	7.6326
4	8.3515	8.35146095	8.3514610	8.3514	7.3577	7.35772548	7.3577422	7.3577
	k=1				k=5			
0	6.9917	6.99174004	6.9917180	6.9917	5.9389	5.93894397	5.9387641	5.9388
1	6.6703	6.67031728	6.6702962	6.6703	5.6659	5.66592012	5.6657485	5.6658
2	6.3895	6.38950303	6.3894829	6.3895	5.4274	5.42739007	5.4272257	5.4272
3	6.1414	6.14140878	6.1413894	6.1414	5.2166	5.21665314	5.2164951	5.2165
4	5.9201	5.92013713	5.9201184	5.9201	5.0287	5.02869994	5.0285476	5.0285

**Table 3:** Effect of temperature change and material gradation on the first nondimensional frequency $\hat{\omega }$ of a simply supported (S–S) FG nanobeam for different nonlocality parameters, under uniform temperature rise (L/h = 20).

μ	ΔT=10 [K]				ΔT=30 [K]				ΔT=60 [K]			
	k=0	k=0.2	k=1	k=5	k=0	k=0.2	k=1	k=5	k=0	k=0.2	k=1	k=5
0	9.64678	7.79268	5.76581	4.68771	9.26239	7.42143	5.41096	4.34763	8.63182	6.80468	4.80700	3.75682
1	9.18597	7.41767	5.48472	4.45692	8.78134	7.02649	5.11022	4.09735	8.11351	6.37147	4.46528	3.46369
2	8.78254	7.08926	5.23844	4.25459	8.35844	6.67876	4.84466	3.87599	7.65378	5.98568	4.15844	3.19839
3	8.42548	6.79843	5.02021	4.07523	7.98241	6.36915	4.60757	3.67792	7.24126	5.63802	3.87936	2.95487
4	8.10640	6.53851	4.82503	3.91468	7.64482	6.09081	4.39384	3.49893	6.86736	5.32149	3.62268	2.72856

**Table 4:** Effect of temperature change and material gradation on the first nondimensional frequency $\hat{\omega }$ of a simply supported (S–S) FG nanobeam for different nonlocality parameters, under linear temperature rise (L/h = 20).

μ	ΔT=10 [K]				ΔT=30 [K]				ΔT=60 [K]			
	k=0	k=0.2	k=1	k=5	k=0	k=0.2	k=1	k=5	k=0	k=0.2	k=1	k=5
0	9.64617	7.79662	5.77176	4.69187	9.45388	7.62133	5.61052	4.53569	9.14754	7.34220	5.35374	4.28687
1	9.18527	7.42169	5.49077	4.46107	8.98312	7.23707	5.32043	4.29579	8.66014	6.94211	5.04802	4.03109
2	8.78183	7.09339	5.24458	4.25872	8.57017	6.89977	5.06547	4.08464	8.23102	6.58940	4.77774	3.80432
3	8.42472	6.80270	5.02645	4.07934	8.20387	6.60036	4.83882	3.89671	7.84887	6.27489	4.53603	3.60093
4	8.10562	6.54285	4.83134	3.91880	7.87580	6.33204	4.63542	3.72782	7.50532	5.99174	4.31774	3.41669

**Table 5:** Effect of temperature change and material gradation on the first nondimensional frequency $\hat{\omega }$ of a simply supported (S–S) FG nanobeam for different nonlocality parameters, under nonlinear temperature rise (L/h = 20).

μ	ΔT=10 [K]				ΔT=30 [K]				ΔT=60 [K]			
	k=0	k=0.2	k=1	k=5	k=0	k=0.2	k=1	k=5	k=0	k=0.2	k=1	k=5
0	9.64611	7.7986	5.7759	4.6957	9.4538	7.6287	5.6252	4.5494	9.1475	7.3591	5.3893	4.3215
1	9.18527	7.4238	5.4953	4.46532	8.9831	7.2443	5.3357	4.3104	8.6602	6.9601	5.0857	4.0678
2	8.78183	7.0956	5.2492	4.2630	8.5702	6.9074	5.0815	4.1001	8.2310	6.6082	4.8176	3.8433
3	8.42472	6.8049	5.0313	4.0839	8.2038	6.6083	4.8556	3.9127	7.8488	6.2947	4.5779	3.6421
4	8.10561	6.5453	4.8363	3.9235	7.8758	6.3404	4.6529	3.7445	7.5053	6.0125	4.3618	3.4598

## Data Availability

The original contributions presented in this study are included in the article. Further inquiries can be directed to the corresponding author.
